# Reviewers in 2023

**DOI:** 10.1098/rsif.2024.0126

**Published:** 2024-03-27

**Authors:** Richard Cogdell

**Affiliations:** *Editor-in-Chief*

I thank all those referees who helped assess submissions to *Journal of the Royal Society Interface* (volume 20) in 2023. The editorial team very much appreciates the time and dedication given by academics to the peer review process and recognizes their efforts. From previous feedback, we know that most reviewers value recognition of their efforts by the journal and through services, such as Publons, which is integrated with *J. R. Soc. Interface*.

To provide an opportunity for recognition, we list the names of all reviewers who provided reports last year (unless they have opted out or not responded). This article is made permanent and citable by its digital object identifier (DOI). We encourage you to quote your contribution in your applications for tenure and grants or other forms of research assessment. Where you have provided it, we have included your ORCID so your contribution can be unambiguously assigned to you.

The team really do appreciate all the work our reviewers do on behalf of the journal, and we look forward to working with you again in 2024.

Oddo C
Engblom S https://orcid.org/0000-0002-3614-1732
Lehmann K https://orcid.org/0000-0003-2690-7332
Imburgia M
Opatowski L https://orcid.org/0000-0001-8348-700X
Gray K
Wood R
Ashmore J
Zenit R https://orcid.org/0000-0002-2717-4954
Machado G
Inoue Y https://orcid.org/0000-0002-1968-8883
Di Puccio F http://orcid.org/0000-0003-4558-1497
Morbiducci U https://orcid.org/0000-0002-9854-1619
Alber M https://orcid.org/0000-0002-7153-1138
Marathe M
Hinow P https://orcid.org/0000-0001-5875-1470
Souron A
Madzvamuse A https://orcid.org/0000-0002-9511-8903
Sheriff RSM
Lacquaniti F
Kuchenbecker K https://orcid.org/0000-0002-5004-0313
Deziel E
Camacho E https://orcid.org/0000-0003-4286-5224
Priede I https://orcid.org/0000-0002-5064-9751
Rainbow M
Imburgia M
Blanke A https://orcid.org/0000-0003-4385-6039
Riisgård HU
Janiga G
Kearns F
Gray K
Meehan C https://orcid.org/0000-0003-0724-8343
Waller L https://orcid.org/0000-0001-5002-8886
Yang Q https://orcid.org/0000-0002-5030-0963
Zheng Y
Putman N https://orcid.org/0000-0001-8485-7455
Gompper G https://orcid.org/0000-0002-8904-0986
Singh A https://orcid.org/0000-0001-9582-670X
Lambropoulou S
Wright A
Reidys C https://orcid.org/0000-0002-6349-4998
Agrawal D
Bablani L
Gonzalez-Parra G https://orcid.org/0000-0001-5847-678X
Schiffer J
Transtrum M
Corbetta A
Schadschneider A
Voloshin A
Casas J https://orcid.org/0000-0003-1666-295X
Baik S
Dahmani J
Lecheval V https://orcid.org/0000-0001-6041-2718
Fröhlich F
Hladky S
Orellana-Rogé L
Singh A https://orcid.org/0000-0001-9582-670X
Ouldridge T
Thomson T
Hsieh F https://orcid.org/0000-0002-9292-6980
Bhushal S
Kim JK https://orcid.org/0000-0001-7842-2172
Brembs B https://orcid.org/0000-0001-7824-7650
Gosler A
Stumpner A
Agrawal D
Winklhofer M https://orcid.org/0000-0003-1352-9723
Han TA https://orcid.org/0000-0002-3095-7714
Boldea O
Steinmann T https://orcid.org/0000-0002-2013-7747
Huppert A
Tawn J
Singha T
Klug A
Velasco Hernández J
Bloomfield-Gadêlha H https://orcid.org/0000-0001-8053-9249
Huang J
Biktashev V https://orcid.org/0000-0001-6617-4677
Dushoff J https://orcid.org/0000-0003-0506-4794
Gonzalez-Parra G https://orcid.org/0000-0001-5847-678X
Schiffer J
Han TA https://orcid.org/0000-0002-3095-7714
Liu L https://orcid.org/0000-0003-0286-8885
Jones R
Scott J https://orcid.org/0000-0003-2971-7673
Huang W https://orcid.org/0000-0002-9016-2665
De Ron A https://orcid.org/0000-0002-8058-6093
Truskey GA
Deshpande A https://orcid.org/0000-0003-2581-2737
Jansen S https://orcid.org/0000-0002-4476-5334
Magal P https://orcid.org/0000-0002-4776-0061
Lohmann K
Holdsworth S
Gölgeli M
Hens N https://orcid.org/0000-0003-1881-0637
Darzi A
Snedden C
Reif J
Parreno J
Hristov J
Svensson O https://orcid.org/0000-0003-3752-3131
Liu H https://orcid.org/0000-0002-8687-3237
Greco G https://orcid.org/0000-0003-3356-7081
Carter C
Di Talia S
Varela J
Parslew B https://orcid.org/0000-0002-0070-488X
Bueno M-A https://orcid.org/0000-0001-5067-217X
Revzen S
McWhorter T https://orcid.org/0000-0002-4746-4975
Hranitz J https://orcid.org/0000-0002-8928-9466
Haydon D https://orcid.org/0000-0002-1240-1886
Lau M
Fearon E https://orcid.org/0000-0001-5574-251X
Wientjes E
Bálint Z
Wang S https://orcid.org/0000-0002-5111-5356
Bhushal S
Holdsworth S
Goergen C
Liao J https://orcid.org/0000-0003-1049-5783
Frachisse JM https://orcid.org/0000-0002-9755-6536
Simons J
Giraldi L https://orcid.org/0000-0003-2684-0203
Alburaki M
Mukherjee N
Lee H-W
Mai C
Cheng J
Velasco Hernández J
Hiroyuki E
Voss-Böhme A https://orcid.org/0000-0001-6510-8694
Carvalho L
Schiele N
Veloz T https://orcid.org/0000-0003-3014-8340
Hinow P https://orcid.org/0000-0001-5875-1470
Tapia Brito E
Pickett B
Zachreson C https://orcid.org/0000-0002-0578-4049
Ódor G
Hu D https://orcid.org/0000-0002-0017-7303
Scott J https://orcid.org/0000-0003-2971-7673
Ladich F
Schwartz J-M https://orcid.org/0000-0002-6472-0184
Seymoure B https://orcid.org/0000-0003-3596-1832
Disney C
Mita T
Weihmann T https://orcid.org/0000-0002-6628-1816
Srinivasan M https://orcid.org/0000-0002-7811-3617
Andersson C https://orcid.org/0000-0003-1457-3240
Williams DL
Shoele K
Mittal R
MacLaurin J
Clemente C https://orcid.org/0000-0001-8174-3890
Fearon E https://orcid.org/0000-0001-5574-251X
Zhang X
Snedden C
Chowell G https://orcid.org/0000-0003-2194-2251
Flores K https://orcid.org/0000-0002-4000-6971
Chaplain M https://orcid.org/0000-0001-5727-2160
Keuschnigg M
Podgornik R
Camacho E https://orcid.org/0000-0003-4286-5224
Cerasuolo M
Huijben S https://orcid.org/0000-0002-3537-1915
Finnilä M
Bianco G https://orcid.org/0000-0002-6144-2959
Danchin A
Janiga G
Mukherjee N
Federle W https://orcid.org/0000-0002-6375-3005
von der Haar T https://orcid.org/0000-0002-6031-9254
Smith A
Tønnesen J
Danet A
Ferreira S https://orcid.org/0000-0001-7159-2769
Horný L
Timashev P
Havurinne V
Leonenko V https://orcid.org/0000-0001-7070-6584
Shnerb N
Batterman R
Dhorda M
Mai C
Pramreiter M
Carvalho L

